# Lung ultrasound outperforms symptom-based screening to detect interstitial lung disease associated with rheumatoid arthritis

**DOI:** 10.1136/rmdopen-2024-005283

**Published:** 2025-02-26

**Authors:** Marie Vermant, Alexandros Kalkanis, Joseph Jacob, Tinne Goos, Emanuela Elsa Cortesi, Heleen Cypers, Nico De Crem, Tine Follet, Stefan Gogaert, Barbara Neerinckx, Veerle Taelman, Nathalie Veyt, Laurens J De Sadeleer, Patrick Verschueren, Wim Wuyts

**Affiliations:** 1 Department of Chronic Diseases and Metabolism, KU Leuven, Leuven, Belgium; 2 Pulmonology, University Hospitals Leuven, Leuven, Belgium; 3 Centre for Medical Image Computing, UCL, London, UK; 4 UCL Respiratory, UCL, London, UK; 5 Rheumatology, University Hospitals Leuven, Leuven, Belgium; 6 Department of Development and Regeneration, KU Leuven, Leuven, Belgium; 7 Rheumatology, KU Leuven University Hospitals Leuven, Leuven, Belgium

**Keywords:** Pulmonary Fibrosis, Arthritis, Rheumatoid, Ultrasonography

## Abstract

**Objectives:**

Interstitial lung disease associated with rheumatoid arthritis (RA-ILD) is linked to high mortality. Currently, effective screening tools are lacking. We assessed the role of symptoms and lung ultrasound (LUS) as potential screening tools.

**Methods:**

116 adult patients with RA presenting to the rheumatology outpatient clinic underwent high-resolution CT (HRCT) scans, pulmonary function tests, LUS (72 zones) and completed a Visual Analogue Scale (VAS) for cough and modified Medical Research Council dyspnoea scale (mMRC). Kruskal-Wallis (KW) tests evaluated the correlation between clinical–radiological HRCT score (no ILD, non-specific abnormalities, subclinical ILD or ILD) and the B-lines on LUS, diffusion capacity (DLCO%pred), forced vital capacity (FVC%pred), VAS Cough and mMRC. Sensitivity and specificity analyses were performed to assess symptom-based questionnaires and the number of B-lines to detect RA-ILD. Area under the receiver operating characteristics (AUROC) for detecting clinical ILD and subclinical ILD were calculated.

**Results:**

In 11.8% of patients, an ILD was detected on HRCT. Additionally, in 5%, a diagnosis of subclinical interstitial lung changes was made. The number of B-lines was most strongly associated with the clinical–radiological score (KW χ²=41.2, p=<0.001). DLCO%pred was also significantly correlated with the clinical–radiological score (KW χ²=27.4, p=<0.001), but FVC%pred, mMRC and VAS cough were not. Cough and dyspnoea only weakly predicted the ILD score in the sensitivity–specificity analyses, while B-lines showed AUROCs>0.9 for predicting subclinical and clinical ILD.

**Conclusion:**

LUS is a promising tool for early detection of RA-ILD, outperforming symptom-based questionnaires or the presence of dyspnoea or cough.

**WHAT IS ALREADY KNOWN ON THIS TOPIC**

- Interstitial lung disease (ILD) is an important extra-articular manifestation in patients with rheumatoid arthritis (RA), linked to high mortality and morbidity. Currently, screening tools are lacking.

**WHAT THIS STUDY ADDS**

- The number of B-lines counted on lung ultrasound (LUS) is more predictive of the presence of ILD than the use of symptom-based questionnaires, such as the modified Medical Research Council dyspnoea scale and the Visual Analogue scale for cough.

**HOW THIS STUDY MIGHT AFFECT RESEARCH, PRACTICE OR POLICY**

- LUS is a potential screening tool for the detection of ILD associated with RA, outperforming symptom-based screening tools.

## Introduction

 Rheumatoid arthritis (RA) is a chronic autoimmune disease that typically presents with symmetric polyarthritis.^1 2^ RA affects between 0.5% and 1% of the population of high-income countries.^3 4^ In addition to the destructive and invalidating musculoskeletal symptoms, it can present with extra-articular manifestations, such as rheumatoid nodules, vasculitis and interstitial lung disease (ILD).^5 6^ The reported prevalence of RA-ILD in the literature is highly variable. However, 1 in 10 patients with RA is estimated to develop a clinically relevant ILD.^7 8^ RA-ILD is an extra-articular manifestation that is associated with high morbidity and mortality.^9^,^11^ Furthermore, even though the overall mortality gap when compared with the general population is closing, the gap persists in patients with RA-ILD.^12^ This means that, currently, pulmonary manifestations of RA and more specifically ILD are driving the increased mortality in patients with RA. Early detection of RA-ILD is essential as it will influence patient counselling, follow-up and treatment decisions. The presence of ILD will affect both disease-modifying antirheumatic drug therapy and the need for antifibrotic treatment. It has been shown that methotrexate has a protective effect on the development of fibrotic ILD, while there are promising preliminary results for abatacept and rituximab in the control of RA-ILD.^13^ Additionally, antifibrotic therapy slows down disease progression but does not reverse any damage in progressive, fibrotic RA-ILD.^14 15^ Earlier detection of fibrotic, progressive ILD leads to earlier treatment initiation and therefore, longer preservation of pulmonary function and improved survival. Hence, screening for ILD in patients with RA is of the utmost importance.

In current clinical practice, patients are screened case-by-case based on risk factors, such as age, male sex and symptoms. When a case of RA-ILD is suspected, the patient is evaluated with a pulmonary function test (PFT) and high-resolution CT scan (HRCT). However, important diagnostic delays often occur, contributing to higher mortality.^16^ Recently, there has been an emerging role for lung ultrasound (LUS) after it had first been studied to detect ILD in patients with systemic sclerosis.^17^,^19^ Ultrasound is a low-cost, dynamic and radiation-free imaging modality. LUS can be used to assess pleural and parenchymal abnormalities, pneumothorax, pleural effusions and the presence of an interstitial syndrome. The interstitial syndrome is characterised by the presence of B-lines. B-lines are vertical, comet-tail-like artefacts that arise from the pleural line and shoot across the entire screen.^20^ They distort the normal appearance of the lung and move synchronously with respiration.

In RA, after several earlier smaller and case-control studies, some recent larger studies have shown very promising results for LUS as a screening tool to detect RA-ILD.^21^,^25^ They indicate that the presence of B-lines on ultrasound is highly sensitive to detect the presence of ILD on HRCT scans. However, there is no consensus yet concerning the ideal protocol and a cut-off for the number of B-lines. Furthermore, there is an ongoing debate about whether LUS should be performed in all patients or only in symptomatic patients.

To the best of our knowledge, ultrasound has not yet been compared with a symptom-based screening system in patients routinely presenting to the rheumatology clinic. This study aimed to assess the role of LUS, using a 72-zone approach, compared with symptom-based questionnaires to detect ILD in patients with RA.

## Methods

### Study design

For a cross-sectional analysis, 116 patients presenting to the outpatient rheumatology clinic were included. All patients were older than 18 years and diagnosed with RA. The diagnosis of RA was made by an expert rheumatologist (PV, BN, VT, HC) guided by the American College of Rheumatology/European Alliance of Associations for Rheumatology classification criteria. Patients were consecutively and randomly recruited from the rheumatology outpatient clinic at University Hospitals Leuven between January 2023 and November 2023.

All patients underwent an HRCT, PFT and LUS. PFT included spirometry and diffusion testing and was performed within 3 months after inclusion. Patients also completed questionnaires concerning their pulmonary and rheumatological symptoms. The questionnaires examined the presence of dyspnoea and cough, and they included the modified Medical Research Council (mMRC) dyspnoea scale and the Visual Analogue Scale for Cough (VAS Cough).

Patients were not involved in trial design.

### Lung ultrasound

LUS was performed using a Philips Lumify portable ultrasound system with a curved 3.5 MHz array probe. A European Respiratory Society-certified examiner (MV) executed all ultrasound examinations. The ultrasound settings were preset to LUS, removing harmonic imaging and lowering the reject post-processing. All examinations were performed in a seated position, using the 72 lung intercostal space protocol, as published by Gargani *et al*.^17^ For each intercostal zone, the number of observed B-lines was counted. If they were confluent, the semiquantitative method as suggested by Gargani and Volpicelli was used.^20 26^ On examination completion, the counted B-lines of the 72 different intercostal zones were added to reach a total count. To aid in this project, an in-house designed Android mapping application was used.^27^ The LUS evaluation process with 72 intercostal zones approximately lasted between 6 and 12 min.

### HRCT scan scoring

**Figure 1:**
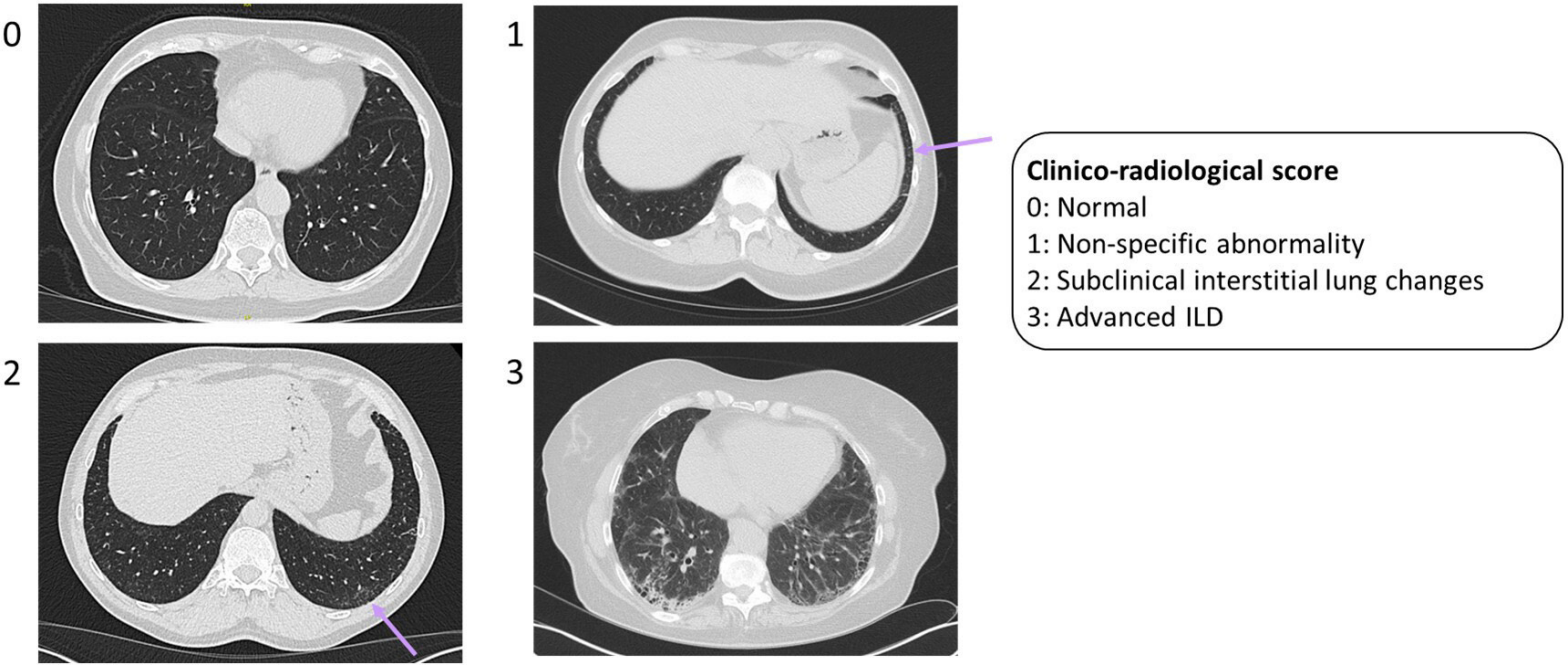
Clinical–radiological score. Reticular lines are visible at both costophrenic angles resulting in a classification of non-specific abnormality and a score of 1 (top right). More widespread ground glass abnormalities and reticular lines are visible at both lung bases suggesting subclinical interstitial changes and a score of 2 (bottom left). Extensive ground glass infiltration is visible in the lower zones, with traction bronchiectasis and honeycomb cysts visible in the subpleural regions of the lower lobes (bottom right).

Non-contrast HRCT or photon counting CT scans (depending on clinical availability) were acquired within 3 months of the ultrasonographic evaluation per clinical protocol at University Hospitals Leuven. Two qualitative CT scores were performed: a clinical–radiological score and a radiological score. The clinical–radiological score (figure 1), performed by an expert pulmonologist (WW), was designed to mimic the advantages of a multidisciplinary team discussion. WW was blinded to the ultrasound findings, but amalgamated medical record data, the in-house radiological assessment, PFTs and the CT images into a 4-point score: 0 (normal), 1 (non-specific abnormalities), 2 (subclinical interstitial lung changes (ILC)) and 3 (advanced ILD). All patients receiving a score of 2 remained in follow-up. Patients assigned a score of 3 were referred for further workup at the outpatient pulmonology department.

As a focused image-based evaluation, a previously described radiological score^28^ (no ILD, non-fibrotic ILC (NFILC), fibrotic ILC (FILC) and advanced ILC (AILC)) was performed by an external specialist chest radiologist (JJ). JJ was blinded to all clinical characteristics, PFTs and ultrasound findings. NFILC represented ground-glass opacities (GGO) or reticulation without any traction bronchiolectasis. FILC represented the combination of traction bronchiolectasis with reticulations or GGOs in a maximum of two lobes. AILC represented the combination of bronchiolectasis with reticulations or GGO in more than two lobes. For all ILCs, a qualitative assessment was added to the score: mild, moderate or severe. The mild score constituted the presence of trivial disease alone and therefore only moderate or severe ILC categories were included in the analysis.

JJ also scored all CT scans using Fleischner Society^29^ criteria for interstitial lung abnormalities (ILA). Although the Fleischner Society^29^ position paper advised against using these criteria in a high-risk population, a lack of alternative criteria required us to consider the Fleischner criteria. However, rather than use the contentious term ILA, we describe ILCs in patients with RA.

### Statistical analysis

The trial was conceptualised as an exploratory study. The baseline characteristics are presented as a median with IQR due to the non-normal distribution of the data. Comparisons between patients referred for a further work-up to confirm the diagnosis of an ILD (clinical–radiological score 3) and the rest of the study cohort were performed with a Fisher’s exact test and Mann-Whitney U test.

Kruskal-Wallis (KW) tests were used to assess the correlation between the HRCT score and the number of B-lines, diffusion capacity % predicted (DLCO%pred), forced vital capacity % predicted (FVC%pred), VAS Cough and mMRC. Intergroup differences were examined using pairwise Wilcoxon rank-sum tests. Correction for multiple testing was performed using the Benjamini and Hochberg false discovery rate method. Sensitivity and specificity analysis for LUS, the presence of dyspnoea and the presence of cough for the detection of HRCT scores were performed. For LUS, a cut-off of 5 B-lines was used. Sensitivity and specificity analyses were performed for both the clinical–radiological and radiological HRCT scores.

Concerning the clinical–radiological scores, area under the receiver operating characteristics (AUROC) for the detection of clinical ILD (score 3) and (sub-)clinical ILD (score 2 or 3) were calculated for the number of B-lines, DLCO%pred, FVC%pred, VAS Cough and mMRC with AUROCs compared using a bootstrapping method.^30^ Data was assumed to be missing at random. Given the exploratory nature of the trial and the fact that missing data was less than 10%, data points with missing data were removed from the analysis.

A total of 110 random 3 s ultrasound clips were scored for the number of B-lines by two independent scorers (MV and AK). An interobserver and intraobserver variability was calculated using Cohen kappa’s inter-rater agreement correlation. Furthermore, an intraclass correlation coefficient was calculated for the number of B-lines.^31^ All statistical analyses were performed in R Studio (V.4.2.2). A significance level of α=0.05 was used for all analyses.

## Results

### Demographics

Of the 116 study patients (table 1), the majority were female, seropositive and ever-smokers. Most patients were in remission, indicated by a median disease activity score 28-C-reactive protein of 2.2. RA duration was highly variable, and almost all patients had received methotrexate at some point during their disease course.

**Table 1:** Baseline characteristics of our study population (n=116)

	n (%)/median (IQR)
Female	74 (63.7)
Seropositive	98 (84.4)
RF positive	81 (69.8)
ACPA positive	87 (75)
Ever smoker	66 (56)
Active smoker	18 (15.5)
Current treatment	
csDMARD	43 (37.1)
csDMARD+bDMARD	46 (39.7)
bDMARD	11 (9.5)
csDMARD+tsDMARD	6 (5.2)
CS+csDMARD	6 (5.2)
CS	1 (0.8)
No therapy	1 (0.8)
Ever use of methotrexate	115 (99.1)
Sonographic features	
> 5 B-lines	56 (48.7)
Pleural abnormalities	46 (40)
Subpleural nodules	16 (13.9)
Age at inclusion	62.9 (17.5)
Age at RA diagnosis	46.8 (20.4)
Disease duration	10.9 (17.6)
DAS28-CRP	2.2 (1.33)
VAS Pain	35 (50)
VAS Fatigue	40 (60)
mMRC	0 (1)
VAS Cough	3 (20)
DLCO%pred	90 (20.5)
FVC%pred	104 (22)
Pack years if ever smoker	11 (20.5)

Figures are shown as percentages for sex, seropositivity, smoking status and treatment and as medians and IQRs for all other variables.

ACPA, anti-citrullinated protein antibody; bDMARD, biological disease-modifying antirheumatic drug; CS, corticosteroids; csDMARD, conventional synthetic disease-modifying antirheumatic drug; DAS28-CRP, disease activity score 28-C-reactive protein; DLCO%pred, predicted diffusion capacity; FVC%pred, predicted forced vital capacity; mMRC, modified Medical Research Council dyspnoea scale; RA, rheumatoid arthritis; RF, rheumatoid factor; tsDMARD, targeted synthetic disease-modifying antirheumatic drug; VAS, Visual Analogue Scale.

109/116 (94%) patients underwent all planned examinations. Six patients missed their planned CT scans and PFTs. When contacted by phone, this was mostly due to the extra time required for these procedures. Additionally, for one patient, the ultrasound results were lost due to an unexpected malfunctioning of our in-house designed application.

### Interobserver and intraobserver variation

Interobserver and intraobserver agreement for the absolute number of B-lines per frame was substantial (K=0.73) and excellent (K=0.87) respectively. The intraclass correlation coefficient for interobserver agreement was 0.93 (95% CI 0.90 to 0.95). The intraobserver and interobserver agreement for the presence/absence of B-lines was excellent (K=0.98) and (K=0.96), respectively.

### Prevalence of ILD

Following the clinical–radiological scoring adopted in our study, a score of 0 was assigned in 52.7% (58) of 110 cases who received a CT scan, a score of 1 in 30% (33), a score of 2 in 5.5% (6) and a score of 3 in 11.8% (13). When using the radiological score, 80.9% (89) of scans showed no ILD, 7.3% (8) had NFILC, 3.6% (4) had FILC and 8.1% (9) had AILC. When using the Fleischner criteria, 18.2% (20) of patients had changes in CT compatible with ILCs (online supplemental figure 1).

Patients who were referred to the pulmonology clinic for further work-up for an underlying, undiagnosed ILD (clinical–radiological score 3) were significantly older and had a significantly lower FVC%pred and DLCO%pred (table 2). No significant differences in respiratory symptoms (VAS Cough and mMRC) were observed.

**Table 2:** Comparison between patients with clinical–radiological RA-ILD (score 3) and patients without clinical–radiological RA-ILD

	RA-ILD (12)N(%)/median (IQR)	RA-noILD (97)N(%)/median (IQR)	P value (Fisher exact, Mann-Whitney U)
Female	7 (58.3)	62 (63.9)	0.75
Seropositive	11 (91.6)	80 (82.5)	0.68
RF positive	10 (83.3)	65 (67)	0.17
ACPA positive	10 (83.3)	70 (72.2)	0.45
Ever smoker	9 (75)	53 (54.6)	0.22
Active smoker	4 (33.3)	12 (12.4)	0.06
Current treatment			0.14
csDMARD	4 (33.3)	36 (37.1)	
csDMARD+bDMARD	2 (16.7)	41 (42.2)	
bDMARD	3 (25)	7 (7.2)	
csDMARD+tsDMARD	1 (8.3)	5 (5.2)	
CS+cDMARD	2 (16.7)	6 (6.2)	
CS	0	1 (1.3)	
No therapy	0	1 (1.3)	
Age at inclusion	72.5 (10)	61.1 (17)	<0.01
Age at RA diagnosis	57.7 (19.5)	45.7 (20.5)	0.04
Median disease duration	17.4 (13.7)	11.0 (19.6)	0.88
DAS28-CRP	2.35 (0.875)	2.1 (1.4)	0.39
VAS Pain	50 (52.5)	30 (50)	0.08
VAS Fatigue	27.5 (46.2)	40 (60)	0.85
mMRC	0.5 (1.25)	0 (1)	0.17
VAS Cough	20 (32.5)	1 (18.8)	0.10
DLCO%pred	63.5 (6.75)	93 (19)	<0.01
FVC%pred	99 (17.5)	104 (22.8)	0.02

Figures are shown as percentages for sex, seropositivity, smoking status and treatment and as medians and IQRs for all other variables. Comparison of RA-ILD and RA-noILD was performed using Fisher’s exact tests (for percentages) and Mann-Whitney U tests (for median values).

ACPA, anti-citrullinated protein antibody; bDMARD, biological disease-modifying antirheumatic drug; CS, corticosteroids; csDMARD, conventional synthetic disease-modifying antirheumatic drug; DAS28-CRP, disease activity score28-C-reactive protein; DLCO%pred, predicted diffusion capacity; FVC%pred, predicted forced vital capacity; ILD, interstitial lung disease; mMRC, modified Medical Research Council dyspnoea scale; RA, rheumatoid arthritis; RF, rheumatoid factor; tsDMARD, targeted synthetic disease-modifying antirheumatic drug; VAS, Visual Analogue Scale.

### Correlation between screening tools and the two HRCT scoring systems

**Figure 2:**
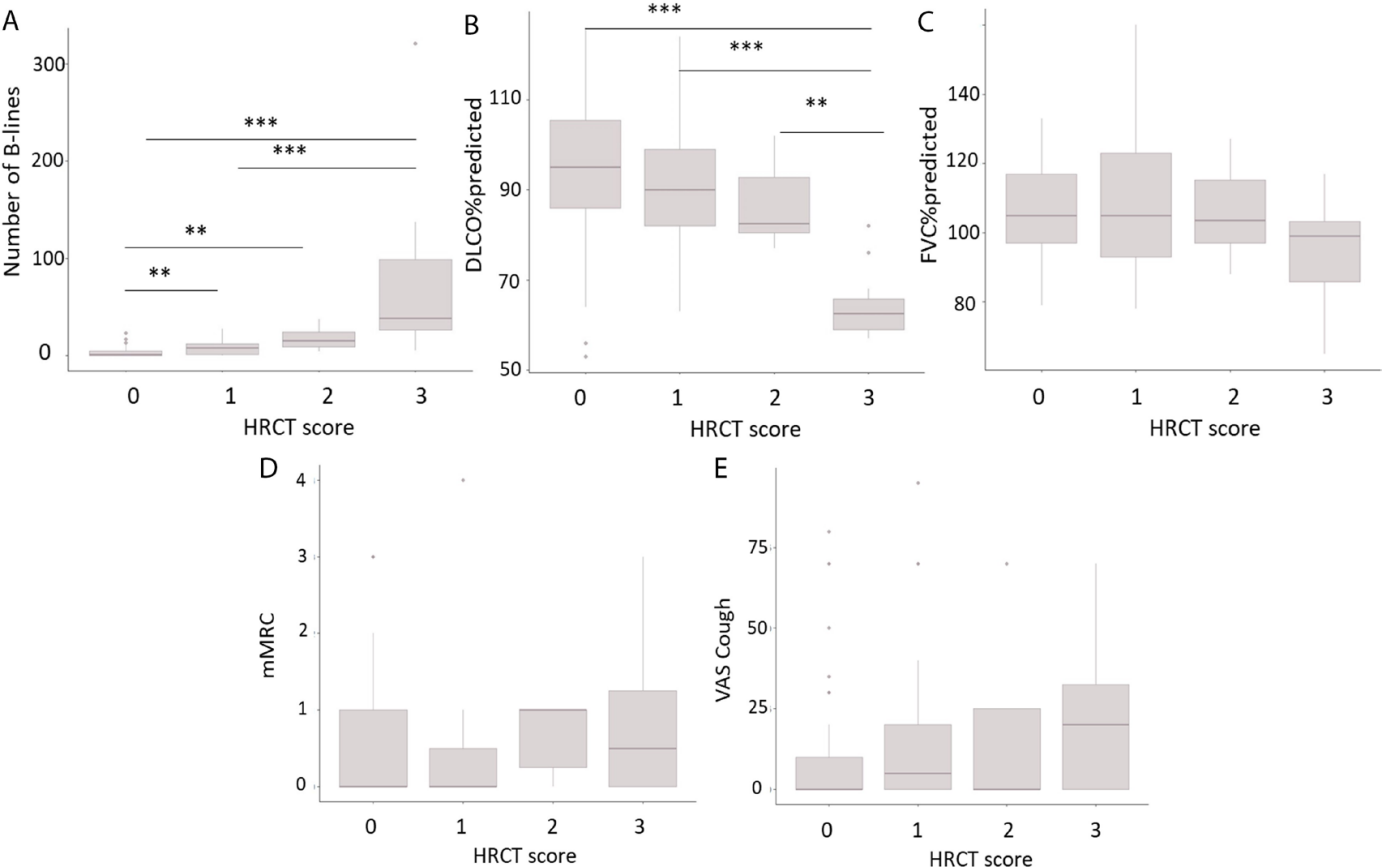
Boxplots showing the number of B-lines, pulmonary function parameters and symptom scores per clinical–radiological HRCT score. Boxplots showing (**A**) the number of B-lines, (**B**) the per cent predicted diffusing capacity of the lungs for carbon monoxide (DLCO), (**C**) the per cent predicted forced vital capacity, (**D**) modified Medical Research Council dyspnoea scale score and (**E**) Visual Analogue Score for Cough per clinical–radiological HRCT score. Differences between groups were assessed using pairwise Wilcoxon rank-sum tests. Significance is indicated by *p<0.05, **p<0.01, ***p<0.001. Correction for multiple testing was performed (Benjamini and Hochberg false discovery rate). DLCO%pred, predicted diffusion capacity; FVC%pred, predicted forced vital capacity; HRCT, high-resolution CT; mMRC, modified Medical Research Council dyspnoea scale; VAS, Visual Analogue Scale.

B-lines (KW χ²=41.2, p<0.001) and DLCO%pred (KW χ²=27.4, p<0.001) were significantly associated with the clinical–radiological score, in contrast to FVC%pred, mMRC and VAS Cough. When using the purely radiological score, this correlation remained significant for B-lines (KW χ²=24.16, p<0.001) and DLCO%pred (KW χ²=19.7, p<0.001) (online supplemental table 1). Figure 2 shows the number of B-lines, DLCO%pred, FVC%pred, mMRC and VAS Cough per clinical–radiological HRCT group as boxplots, highlighting intergroup differences for the number of B-lines and DLCO%pred. Online supplemental figure 2 shows the boxplots of the aforementioned parameters for the purely radiological score.

### Sensitivity and specificity of different screening tools

Using a cut-off of 5 B-lines, the sensitivity for both radiological definitions of advanced ILD (clinical–radiological score=3 or radiological AILC) was 100%. Specificity was 54% for AILC and 56% for the clinical–radiological score 3. The sensitivity for the presence of ILCs (clinical–radiological score>1; radiological NFILC, FILC, and AILC; Fleischner score ILC presence) varied between 90% and 100% depending on the definition used. The negative predictive value for the presence of ILCs was between 0.96 and 0.98 depending on the definitions used and 1 for the presence of ILD. The complete sensitivity–specificity analysis can be found in table 3.

**Table 3:** Sensitivity and specificity analysis for a cut-off of 5 B-lines for ILD assessed using three different scoring systems: clinical–radiological score, purely radiological score and Fleischner score

	CT score 2/3	CT score 3	NFILC/FILC/AILC	FILC/AILC	AILC	Fleischner ILC
Sensitivity	94.44	100.00	95.24	100.00	100	90.00
Specificity	58.24	55.67	60.23	56.25	54	58.43
PPV	0.31	0.22	0.36	0.24	0.16	0.33
NPV	0.98	1.00	0.98	1.00	1	0.96
LR+	2.26	2.26	2.39	2.29	2.17	2.16
LR−	0.10	0	0.08	0	0	0.17

AILC, advanced interstitial lung changes; FILC, fibrotic interstitial lung changes; Fleischner ILC, interstitial lung changes when applying the criteria of the 2020 Fleischner criteria position paper; ILD, interstitial lung disease; LR, likelihood ratio; NFILC, non-fibrotic interstitial lung changes; NPV, negative predictive value; PPV, positive predictive value; score 3, clinical ILD; score 2/3, subclinical and clinical ILD.

Table 4 shows the sensitivity and specificity for the presence of dyspnoea and cough for the different definitions of ILD and ILC. The sensitivity and specificity for the presence of dyspnoea to detect the clinical–radiological score 3 were 62% and 50%, respectively. For the presence of cough, the sensitivity and specificity to detect score 3 were 54% and 66%, respectively.

**Table 4:** Sensitivity and specificity analysis using the presence of dyspnoea/cough assessed using three different scoring systems: clinical–radiological score, purely radiological score and Fleischner score

Presence of dyspnoea (yes/no)				FILC/AILC	AILC	Fleischner ILC
	CT score 2/3	CT score 3	NFILC/FILC/AILC			
Sensitivity	52.63	61.54	47.62	61.54	66.67	50.00
Specificity	65.88	50.00	65.06	65.93	50.00	65.48
PPV	0.26	0.21	0.26	0.21	0.15	0.26
NPV	0.86	0.86	0.83	0.92	0.92	0.85
LR+	1.54	1.23	1.36	1.81	1.33	1.45
LR−	0.72	0.77	0.81	0.58	0.67	0.76

Presence of cough (yes/no)				FILC/AILC	AILC	Fleischner ILC
	CT score 2/3	CT score 3	NFILC/FILC/AILC			
Sensitivity	50.00	53.85	50.00	41.67	44.44	50.00
Specificity	65.88	65.56	66.27	63.74	63.83	66.27
PPV	0.24	0.18	0.26	0.13	0.11	0.26
NPV	0.86	0.91	0.85	0.89	0.92	0.85
LR+	1.47	1.56	1.48	1.15	1.23	1.48
LR−	0.76	0.70	0.75	0.92	0.87	0.75

AILC, advanced interstitial lung changes; FILC, fibrotic interstitial lung changes; Fleischner ILC, interstitial lung changes when applying the criteria of the 2020 Fleischner criteria position paper; LR, likelihood ratio; NFILC, non-fibrotic interstitial lung changes; NPV, negative predictive value; PPV, positive predictive value; score 3, clinical ILD; score 2/3, subclinical and clinical ILD.

DLCO%pred, when using a cut-off of 80%, was found to be a highly specific tool for the detection of clinical (82.5–85.1%) and subclinical ILD (82.6–85.2%), with a high sensitivity for clinical ILD (88.9–91.7%). It was not as sensitive for the detection of subclinical ILD (50–66.7%), especially when using the ILC score based on the Fleischner 2020 position paper (50%). The complete sensitivity and specificity analysis for the clinical–radiological score and purely radiological score are shown in online supplemental table 2.

### Area under the receiver operator curve

**Figure 3:**
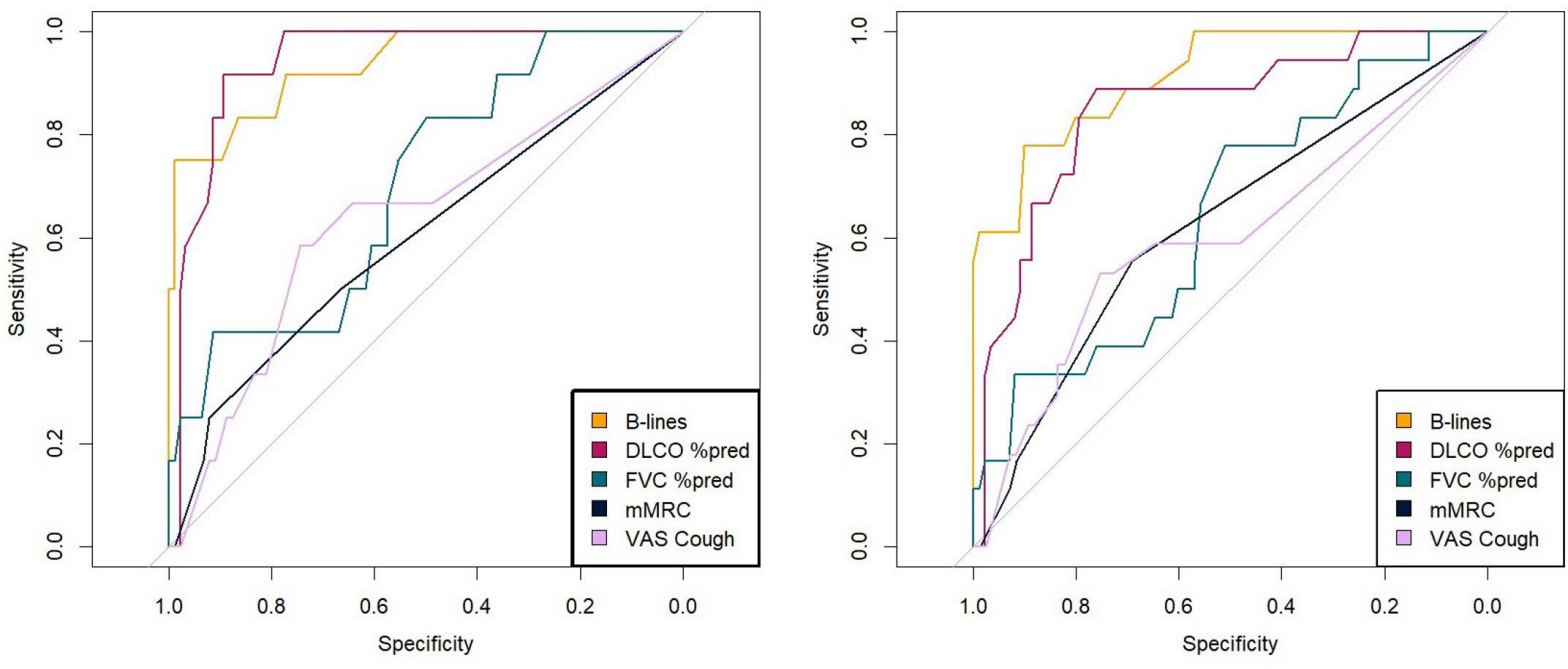
ROC curves for the detection of clinical–radiological score 3 (left) and clinical–radiological scores 2 and 3 (right) based on LUS versus lung function testing and clinical symptoms. B-lines, the number of B-lines counted on lung ultrasound using a 72-zone protocol; DLCO%pred, predicted diffusion capacity; FVC%pred, predicted forced vital capacity; LUS, lung ultrasound; mMRC, modified Medical Research Council dyspnoea scale; ROC, receiver operating characteristics; VAS, Visual Analogue Scale.

The number of B-lines showed AUROCs>0.9 for predicting both a clinical–radiological score of 3 and ILD score≥2 (figure 3 and table 5). DLCO%pred also had an AUROC>0.9 for predicting a clinical–radiological score of 3. When comparing the AUROCs for a clinical–radiological score of 3, B-lines outperformed FVC (p=0.007), mMRC (p<0.001) and VAS Cough (p<0.001). For a clinical–radiological score of 2 or 3, the results were maintained. Similar results were obtained for the AUROC curves for the purely radiological scores and the Fleischner criteria (online supplemental figures 3 and 4 and tables 2 and 3).

**Table 5:** Areas under the operator curve for multiple screening tools

	CT score 3 (clinical ILD)	CT score 2/3
B-lines	0.936 (0.861–1)	0.914 (0.845–1)
mMRC	0.603 (0.434–0.772)	0.624 (0.492–0.755)
VAS Cough	0.637 (0.460–0.815)	0.600 (0.441–0.759)
FVC%pred	0.702 (0.547–0.857)	0.643 (0.502–0.784)
DLCO%pred	0.942 (0.895–0.990)	0.849 (0.749–0.950)

B-lines, the number of B-lines counted on lung ultrasound using a 72-zone protocol; DLCO%pred, predicted diffusion capacity; FVC%pred, predicted forced vital capacity; ILD, interstitial lung disease; mMRC, modified Medical Research Council dyspnoea scale; VAS, Visual Analogue Scale.

## Discussion

We detected clinically relevant ILD in 11.8% and subclinical ILCs in 5.5% of patients in our cohort of patients with well-controlled RA with a heterogeneous disease duration. Patients with RA-ILD were typically older than patients with RA without ILD. A LUS scanning protocol encompassing 72 intercostal zones was used to assess the total number of B-lines. When comparing AUROCs, LUS significantly outperformed symptom-based scores.

Currently, there is no standardised way to determine important ILCs in a high-risk population. As noted earlier, the Fleischner criteria for ILA are not supposed to be used in this context, yet no alternative is present. Both the clinical–radiological score and purely radiological score were able to identify patients with relevant ILCs, which is reflected by the reduced diffusion capacity in these patients. For the clinical–radiological score, the pulmonologist was not blinded for all clinical parameters due to the important clinical implications. While the clinical–radiological score might be advantageous as it encompasses more than the radiological findings and serves as a surrogate for a multidisciplinary discussion, it might also have introduced some bias. To address the existence of potential bias, we validated our results with a purely radiological score, where the radiologist was blinded for all other parameters. This radiological score fully replicated the results, further strengthening our conclusion. In the absence of a predefined consensus, we feel that the use of multiple CT scoring systems in our comparison provides more information on the diagnostic accuracy of LUS. This further strengthens its position in future research and a clinical setting. Given the very high negative predictive value of ultrasound to rule out ILCs, when using the clinical–radiological, radiological and Fleischner criteria, we believe that LUS represents a good screening tool to detect patients in need of a pulmonological workup for an undiagnosed ILD. Caution is, however, warranted for an immediate clinical implementation due to the relatively low positive predictive value of 33%, which might have substantial practical implications. This relatively low positive predictive value and the corresponding low specificity of LUS can be explained by the broad range of underlying health problems associated with the presence of B-lines. B-lines can be present in underlying cardiac disease, viral infections, pneumonia, pneumonitis, pleural disease, atelectasis and small deaeration areas.^2032^,^34^ A limited number of these artefacts can also be a normal finding in elderly patients.^35^ Additionally, closely resembling B-lines, Am-lines have been described in emphysema, which can influence the interpretation.^25^ The setting of our cut-off to 5 B-lines, which is relatively low, influences our findings as lower cut-offs are associated with higher sensitivity and lower specificity.^36^ The high sensitivity is important in a screening tool, to be able to rule out the presence of an underlying ILD.

Ultrasound has some important advantages over other possible diagnostic tools. This is reflected in the amount of missing CTs and PFTs compared with the number of missing ultrasound scans. Returning to the clinic for further testing and the additional time needed for technical examinations felt like a burden for patients, especially compared with LUS, which was easily combined with their routine visits. Moreover, it is a dynamic tool, and it is associated with lower costs than HRCT or PFT. The rheumatological patients were also very familiar with the concept of ultrasound. Furthermore, the lack of radiation is very favourable, especially in a younger population.

The interobserver and intraobserver agreement for the number of B-lines was substantial, suggesting that with ample training, ultrasound can be a reliable tool with good reproducibility. These results were in line with findings from previous studies on the detection of an interstitial syndrome in patients with COVID-19 and systemic sclerosis.^37 38^ The consistency with which B-lines are detected in different clinical settings further supports the increased use of LUS in clinical practice.

Alongside several strengths, our study is not without limitations. First, our LUS protocol is quite extensive as it comprises 72 intercostal zones. However, the entire scanning protocol only takes between 6 and 12 min if performed by an experienced operator. Furthermore, when translating this protocol to a pragmatic, clinical setting, one could argue to stop the protocol on identifying 5 B-lines. This would lead to even shorter examination times.

Second, ultrasound is an operator-dependent technique which might affect the reproducibility of our results and the implementation of LUS into clinical practice. Nevertheless, our high interobserver agreement for the number of B-lines further supports our claim that LUS is a promising screening tool for the detection of RA-ILD when the operator is adequately trained.

Additionally, this is a single-centre, exploratory study with approximately 6% missing data. This might have introduced some selection bias. Nevertheless, the rheumatology department of the University Hospital Leuven fulfils a role both in secondary and tertiary care, meaning that part of our patients is referred directly by their general practitioner for consultation, and part is referred by rheumatologists for specialised advice.

To conclude, LUS is a feasible and reliable screening tool. It improves on the low sensitivity and specificity of symptom scores to detect clinically relevant and subclinical RA-ILD. While further collaborative research is necessary to define consensus protocols to identify subjects for screening, determine appropriate cut-offs for the number of B-lines and define optimal screening intervals, our findings suggest that LUS could have a transformative role in the early detection of RA-ILD.

## Supplementary material

10.1136/rmdopen-2024-005283 online supplemental file 1

## Data Availability

Data are available upon reasonable request.
