# Supplementary material for: Lung ultrasound outperforms symptom-based screening to detect interstitial lung disease associated with rheumatoid arthritis
Source: RMD Open. 2025 Feb 26;11(1):e005283. doi: 10.1136/rmdopen-2024-005283 (PMC12083264; doi:10.1136/rmdopen-2024-005283)
Supplement: online supplemental file 1 [file rmdopen-11-1-s001.docx]

# Lung ultrasound outperforms symptom-based screening to detect interstitial lung disease associated with rheumatoid arthritis.

## Supplementary material

### Supplementary Figure 1. Patients per score


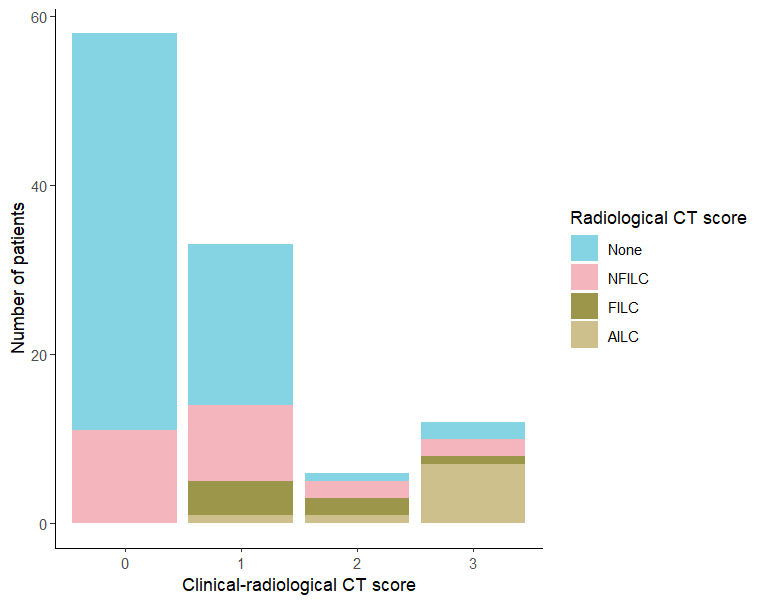

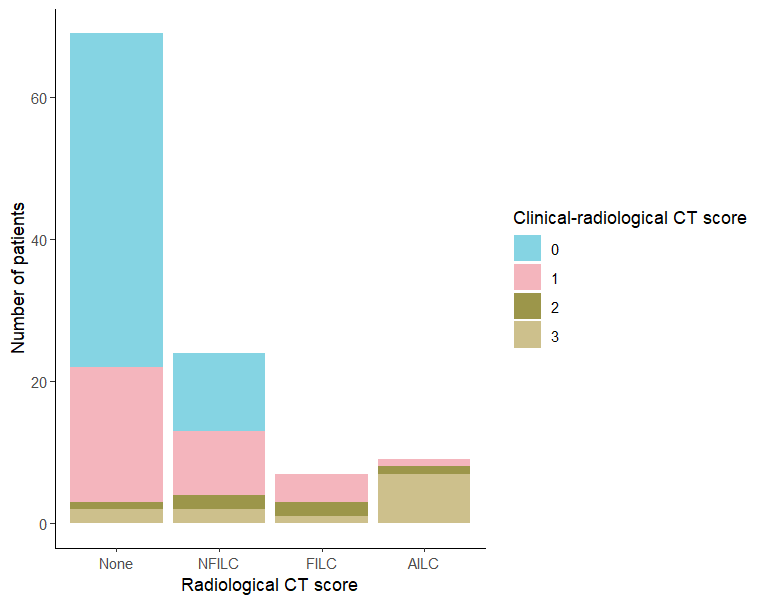


Left: Number of patients per clinical-radiological score, colored based on the purely radiological score. Right: Number of patients for each purely radiological score, colored based on their clinical-radiological score

### Supplementary Table 1. Kruskall Wallis Chi-Squared test for the purely radiological score (None, NFILC, FILC, AILC) and possible screening tools.

|  | KW Chi2 | p |
| --- | --- | --- |
| B-lines | 24,16 | <0,001 |
| DLCO%pred | 19,07 | <0,002 |
| FVC%pred | 5,78 | 0,12 |
| mMRC | 3,75 | 0,29 |
| VAS Cough | 1,50 | 0,68 |

*B-lines= the number of B-lines counted on lung ultrasound using a 72-zone protocol, VAS = visual analogue scale, mMRC = modified medical research council dyspnea scale, DLCO%pred= predicted diffusion capacity, FVC%pred= predicted forced vital capacity*

### Supplementary Figure 2. Boxplots, showing the number of B-lines, predicted DLCO, predicted FVC, mMRC and VAS Cough stratified by the purely radiological score


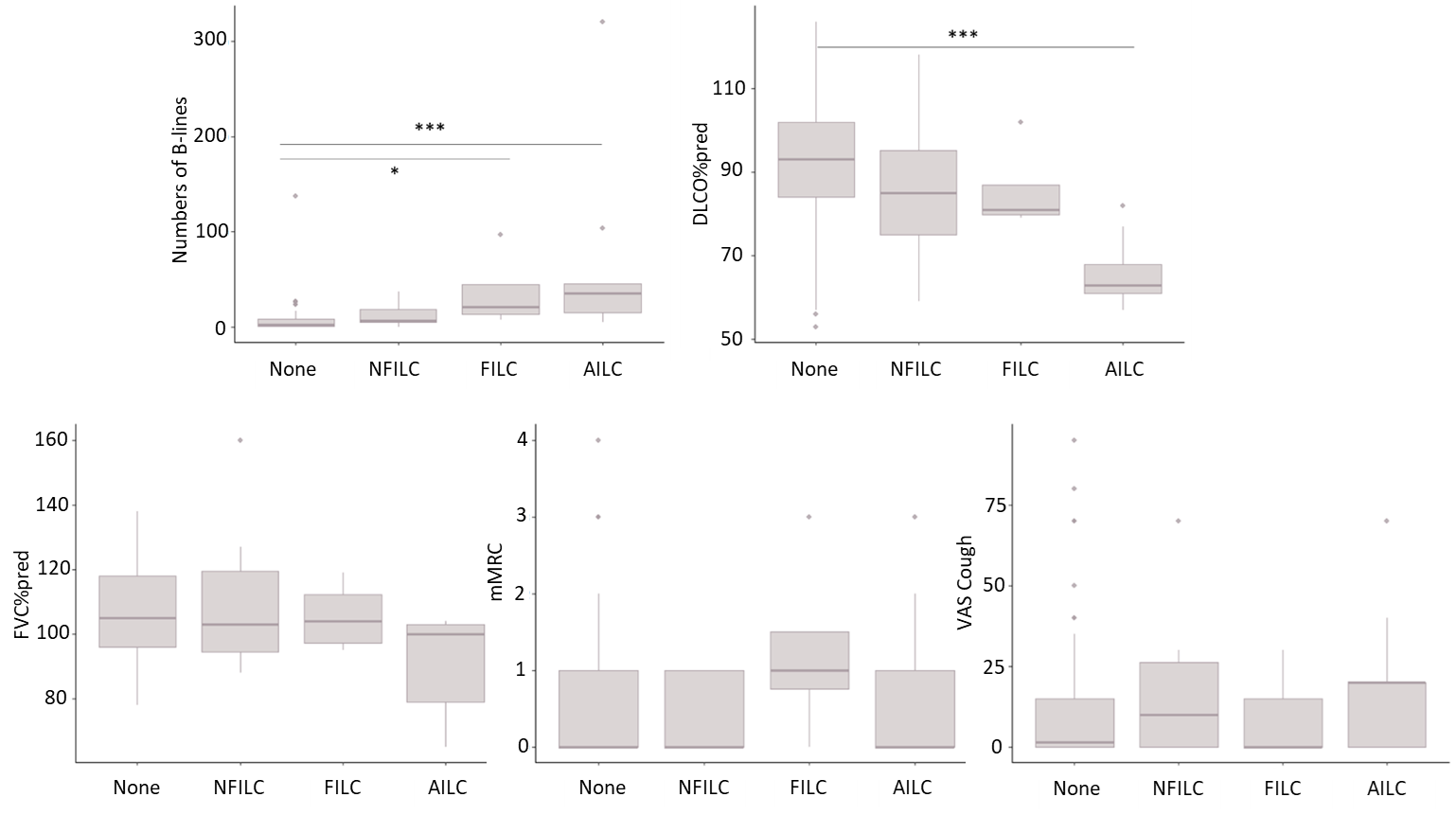


*B-lines= the number of B-lines counted on lung ultrasound using a 72-zone protocol, VAS = visual analogue scale, mMRC = modified medical research council dyspnea scale, DLCO%pred= predicted diffusion capacity, FVC%pred= predicted forced vital capacity, NFILC= non fibrotic interstitial lung changes, FILC= fibrotic interstitial lung changes, AILC = advanced interstitial lung changes, Fleischner ILC = interstitial lung changes when applying the criteria of the 2020 Fleischner criteria position paper*

### Supplementary Table 2. Sens/Spec analyses for DLCO < 80% to predict ILD assessed using three different scoring systems: clinical-radiological score, purely radiological score and Fleischner score.

| **DLCO <80%** | | | | | | |
| --- | --- | --- | --- | --- | --- | --- |
|  | **CT score 2/3** | **CT score 3** | **NFILC/FILC/AILC** | **FILC/AILC** | **AILC** | **Fleischner ILC** |
| Sensitivity | 66,67 | 91,67 | 57,14 | 69,23 | 88,89 | 50,00 |
| Specificity | 85,23 | 85,11 | 84,71 | 82,80 | 82,47 | 82,56 |
| PPV | 0,48 | 0,44 | 0,48 | 0,36 | 0,32 | 0,40 |
| NPV | 0,93 | 0,99 | 0,89 | 0,95 | 0,99 | 0,88 |
| LR+ | 4,51 | 6,15 | 3,74 | 4,02 | 5,07 | 2,87 |
| LR- | 0,39 | 0,10 | 0,51 | 0,37 | 0,13 | 0,61 |

*PV= positive predictive value, NPV= negative predictive value, LR= likelihood ratio, score 2/3= subclinical and clinical ILD, score 3 = clinical ILD, NFILC= non fibrotic interstitial lung changes, FILC= fibrotic interstitial lung changes, AILC = advanced interstitial lung changes, Fleischner ILC = interstitial lung changes when applying the criteria of the 2020 Fleischner criteria position paper*

### Supplementary Figure 3. ROCs for interstitial lung changes based on the purely radiological score


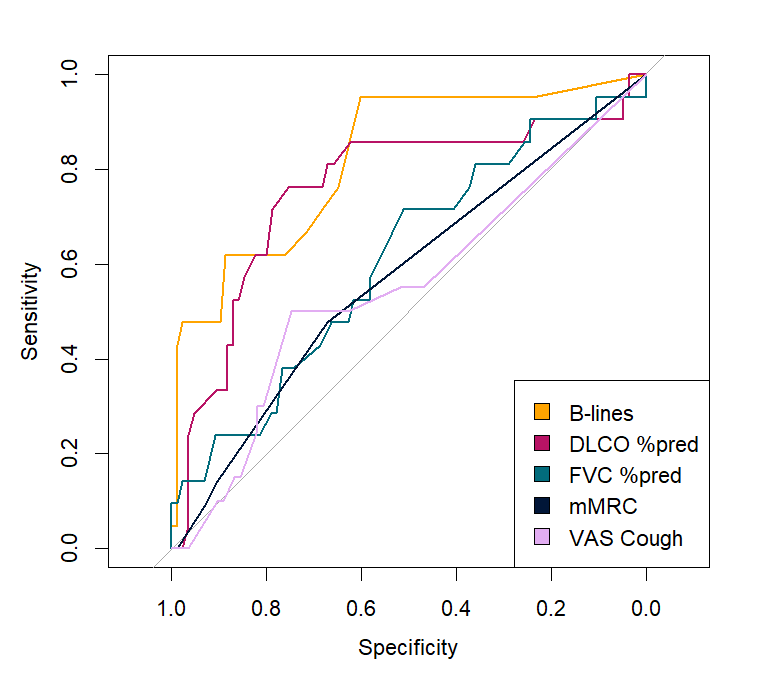

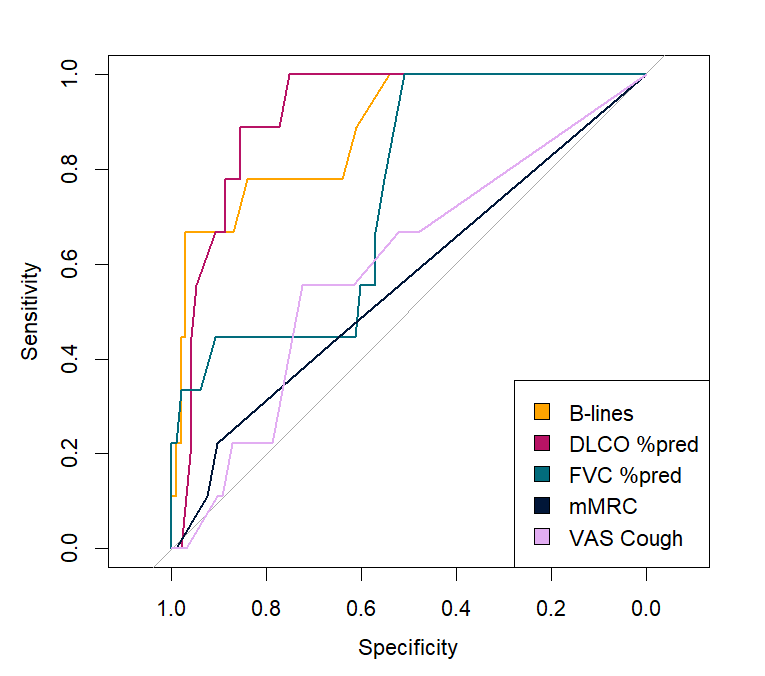

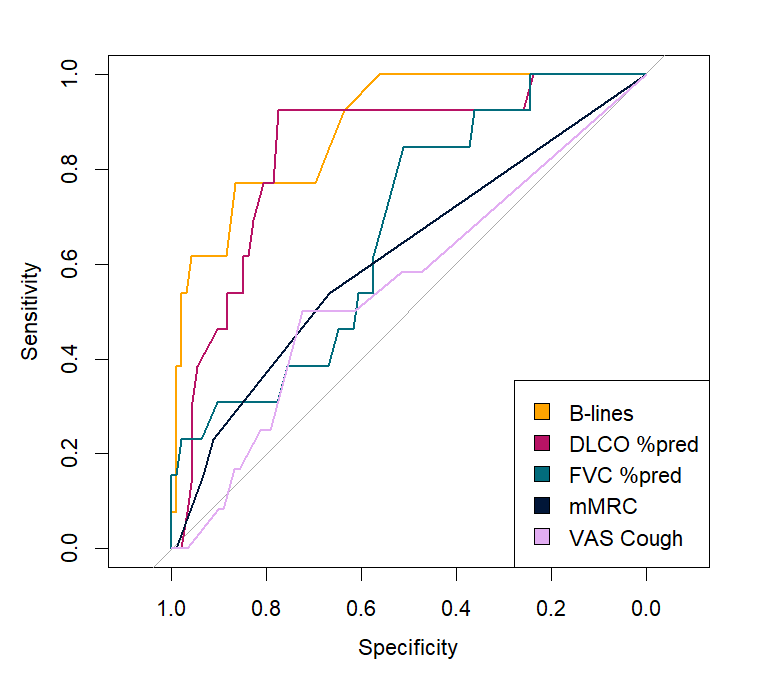


**B**

**A**

**C**

1. **ROCs for Advanced Interstitial Lung Changes (AILC) per diagnostic tool (purely radiological score).**
2. **ROCs for Advanced Interstitial lung Changes (AILC) and Fibrotic Interstitial Lung Changes (FILC) per diagnostic tool (purely radiological score).**
3. **ROCs for Advanced Interstitial lung Changes (AILC), Fibrotic Interstitial Lung Changes (FILC), and Non-Fibrotic Interstitial Lung Changes (NFILC) per diagnostic tool (purely radiological score)**

*B-lines= the number of B-lines counted on lung ultrasound using a 72-zone protocol, VAS = visual analogue scale, mMRC = modified medical research council dyspnea scale, DLCO%pred= predicted diffusion capacity, FVC%pred= predicted forced vital capacity*

### Supplementary Table 2. AUROCs including confidence intervals for the purely radiological score

|  | AILC | AILC/FILC | AILC/FILC/NFILC |
| --- | --- | --- | --- |
| B-lines | 0,883 (0,769-0,996) | 0,889 (0,803-0,976) | 0,823 (0,720-0,927) |
| DLCO%pred | 0,916 (0,854 -0,978) | 0,840 (0,725-0,954) | 0,758 (0,625 - 0,891) |
| FVC%pred | 0,745 (0,590-0,901) | 0,673 (0,527 - 0,820) | 0,609 (0,470 -0,747) |
| mMRC | 0,561 (0,368 -0,754) | 0,615 (0,457 -0,774) | 0,573 (0,449 -0,696) |
| VAS cough | 0,597 (0,401 - 0793) | 0,556 (0,380 - 0,732) | 0,552 (0,408 - 0,696) |

*B-lines= the number of B-lines counted on lung ultrasound using a 72-zone protocol, VAS = visual analogue scale, mMRC = modified medical research council dyspnea scale, DLCO%pred= predicted diffusion capacity, FVC%pred= predicted forced vital capacity, NFILC= non fibrotic interstitial lung changes, FILC= fibrotic interstitial lung changes, AILC = advanced interstitial lung changes*

### Supplementary Figure 4. ROCs for the detection of interstitial lung changes when applying the 2020 Fleischner position paper criteria per diagnostic tool


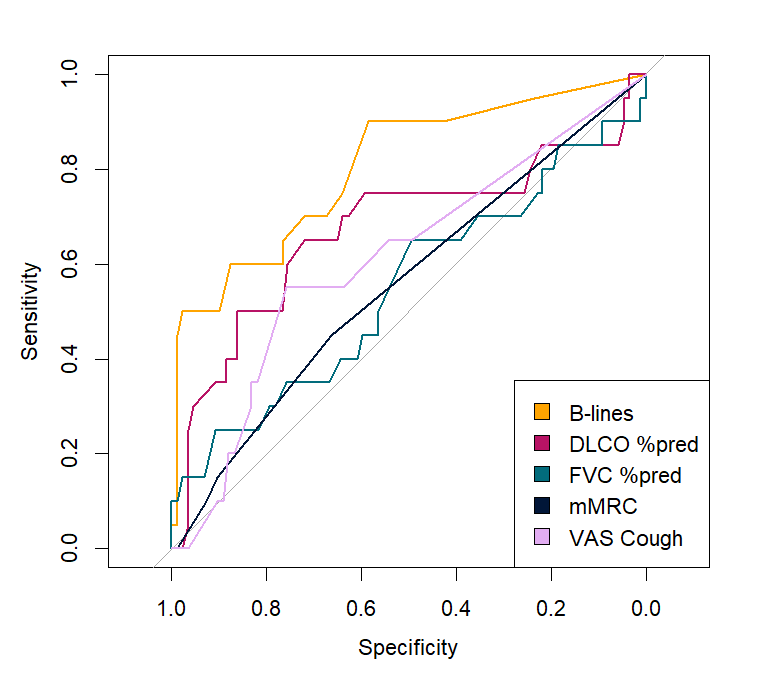


*B-lines= the number of B-lines counted on lung ultrasound using a 72-zone protocol, VAS = visual analogue scale, mMRC = modified medical research council dyspnea scale, DLCO%pred= predicted diffusion capacity, FVC%pred= predicted forced vital capacity*

### Supplementary table 3. AUROCs for interstitial lung changes when applying the 2020 Fleischner position paper criteria

|  | Fleischner |
| --- | --- |
| B-lines | 0,806 (0,690 -0,922) |
| DLCO%pred | 0,673 (0,515 - 0,831) |
| FVC%pred | 0,547 (0,390 - 0,703) |
| mMRC | 0,559 (0,432 -0,685) |
| VAS cough | 0,612 (0,473 -0,750) |

*B-lines= the number of B-lines counted on lung ultrasound using a 72-zone protocol, VAS = visual analogue scale, mMRC = modified medical research council dyspnea scale, DLCO%pred= predicted diffusion capacity, FVC%pred= predicted forced vital capacity*
